# Malaria elimination challenges in Mesoamerica: evidence of submicroscopic malaria reservoirs in Guatemala

**DOI:** 10.1186/s12936-016-1500-6

**Published:** 2016-08-30

**Authors:** Shirley Evelyn Lennon, Adolfo Miranda, Juliana Henao, Andres F. Vallejo, Julianh Perez, Alvaro Alvarez, Myriam Arévalo-Herrera, Sócrates Herrera

**Affiliations:** 1 Malaria Vaccine and Drug Development Center (MVDC), Cali, Colombia; 2 Centro Nacional de Epidemiología (CNE), Guatemala City, Guatemala; 3 Caucaseco Scientific Research Center (CSRC)/Centro Latino Americano de Investigación en Malaria (CLAIM), Cali, Colombia; 4 Facultad de Salud, Universidad del Valle, Cali, Colombia

**Keywords:** Malaria, *Plasmodium vivax*, Prevalence, Asymptomatic, Submicroscopic, Gametocytes, Guatemala, Mesoamerica

## Abstract

**Background:**

Even though malaria incidence has decreased substantially in Guatemala since 2000, Guatemala remains one of the countries with the highest malaria transmission in Mesoamerica. Guatemala is committed to eliminating malaria as part of the initiative ‘Elimination of Malaria in Mesoamerica and the Island of Hispaniola’ (EMMIE); however, it is still in the control phase. During the past decade, the government strengthened malaria control activities including mass distribution of long-lasting insecticide-impregnated bed nets, early diagnosis and prompt treatment. This study aimed to determine the prevalence of malaria, including gametocytes, in three areas of Guatemala using active case detection (ACD) and quantitative polymerase chain reaction (qPCR).

**Methods:**

Cross-sectional surveys were conducted in three departments with varying transmission intensities: Escuintla, Alta Verapaz and Zacapa. Blood samples from 706 volunteers were screened for malaria using microscopy and qPCR which was also used to determine the prevalence of gametocytes among infected individuals. Results were collected and analysed using REDCap and R Project, respectively.

**Results:**

Malaria was diagnosed by microscopy in only 2.8 % (4/141) of the volunteers from Escuintla. By contrast, qPCR detected a prevalence of 7.1 % (10/141) in the same volunteers, 8.4 % (36/429) in Alta Verapaz, and 5.9 % (8/136) in Zacapa. Overall, 7.6 % (54/706) of the screened individuals were positive, with an average parasitaemia level of 40.2 parasites/μL (range 1–1133 parasites/μL) and 27.8 % carried mature gametocytes. Fifty-seven percent (31/54) of qPCR positive volunteers were asymptomatic and out of the 42.6 % of symptomatic individuals, only one had a positive microscopy result.

**Conclusions:**

This study found a considerable number of asymptomatic *P. vivax* infections that were mostly submicroscopic, of which, approximately one-quarter harboured mature gametocytes. This pattern is likely to contribute to maintaining transmission across the region. Robust surveillance systems, molecular diagnostic tests and tailored malaria detection activities for each endemic site may prove to be imperative in accelerating malaria elimination in Guatemala and possibly across all of Mesoamerica.

**Electronic supplementary material:**

The online version of this article (doi:10.1186/s12936-016-1500-6) contains supplementary material, which is available to authorized users.

## Background

**Fig. 1:**
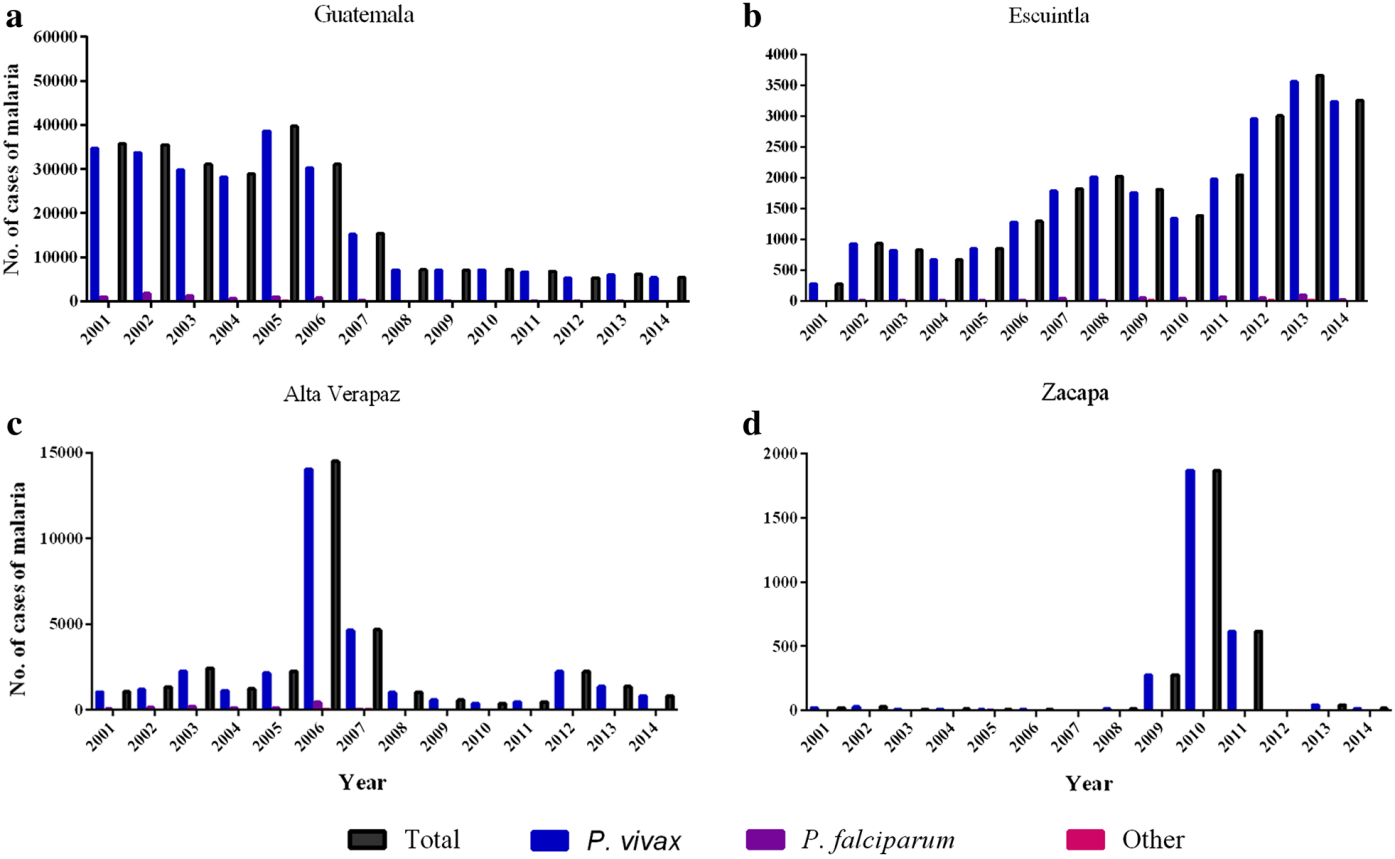
Malaria cases in recent years (2000–2015). **a** Guatemala, **b** Escuintla, **c** Alta Verapaz and **d** Zacapa. Data from the Guatemala MOH (2015), WHO (2014) and the Pan American Health Organization (PAHO) (PAHO data currently available up to 2013). Malaria cases per *Plasmodium* species not available for Guatemala in 2015

Malaria still represents a major global public health issue despite the significant reduction of cases during the past decade [1]. In the 2015 World Malaria Report, the World Health Organization (WHO) estimated 214 million cases and 438,000 deaths worldwide [1]. The seven countries in Central America, with a total of 164 million inhabitants, reported ~10,000 malaria cases, 2.6 % of the total cases in the Americas (~390,000 cases); although it is likely that these figures underestimate the true numbers due to underdiagnosing and underreporting. The incidence of malaria in Guatemala has decreased by 90.5 % since 2000 and the majority of infections are caused by *Plasmodium vivax* (98.0 %), with *Plasmodium falciparum* contributing the remaining 2.0 % [1] (Fig. 1a).

In recent years, the Americas region has shown remarkable progress in malaria control with a reduction of ~67.5 % in cases between 2000 and 2014 [1]. Seven countries in the Americas are now in the elimination phase; Argentina, Belize, Costa Rica, Ecuador, El Salvador, Mexico and Paraguay [1]. Despite this significant regional reduction in malaria burden, which was most remarkable in the Mesoamerican region, several countries including Panama, Nicaragua, Honduras and Guatemala still maintain significant transmission [1].

Guatemala, with an estimated population of 16 million and over 20 ethnic groups and languages spoken, has geographic conditions that favour malaria transmission in most of the country. Malaria transmission in Guatemala is perennial, with most cases occurring during the rainy season, which lasts from May until the end of October. May is also the hottest month of the year. In 2014, Guatemala reported 4931 malaria cases that corresponded to ~47.5 % of the malaria burden in Central America [1]. Fewer cases were reported in 2015 (2634) [Guatemalan Ministry of Health (MOH) surveillance data 2015], however, at the time of writing, the total number of cases in Central America was not available for this year. The Cuchumatanes and Sierra Madre mountain ranges divide Guatemala into three main regions; the most populated area in the central highlands, the costal tropical regions on the Pacific and Caribbean coasts and the northern tropical lowlands in the Petén region. Significant control efforts by the National Malaria Control Programme (NMCP) and the Global Fund to Fight AIDS, Tuberculosis and Malaria (GFATM) have helped to reduce the incidence of malaria over the past decade. However, many challenges remain and factors such as the tropical and humid climate, poverty, lack of education and poor diagnostics and adherence to treatment all contribute to the maintenance of malaria in the country.

In 2005, the NMCP, with support of the GFATM, launched an initiative to prevent and control malaria in Guatemala. Activities included strengthening the malaria surveillance system and prompt diagnosis and treatment. However, by 2009 malaria was endemic in 27 of the 29 administrative health areas and a further GFATM sponsored initiative began [2]. An active program of distribution of long-lasting insecticide-treated nets (LLIN), vector control activities, prompt diagnosis and treatment, among other activities, took place alongside a significant reduction in malaria cases [2]. From 2000 to 2014 the Annual Parasite Index fell from 4.4 to 0.3 per 1000 inhabitants [1, 3].

As the number of malaria cases is falling worldwide, more asymptomatic, submicroscopic malaria reservoirs are being found [4–6], which require detection strategies different to those for symptomatic, microscopic infections. It is known that asymptomatic cases represent parasite reservoirs that contribute to malaria transmission [5, 7], but to what degree is unknown [6] and depends on various host and parasite factors [5, 7, 8]. Among these, the production of mature gametocytes in adequate densities is necessary to transfer the parasite from humans to mosquitoes [7, 9]. Other factors include host immune responses and the duration of the infection [4, 10]. Guatemala currently participates in the initiative Elimination of Malaria in Mesoamerica and the Island of Hispaniola (EMMIE initiative) sponsored by the GFATM, whose activities are oriented to facilitate regional efforts towards malaria elimination [11]. However, since participating countries have only recently begun these efforts, there is a knowledge gap regarding how these strategies should be implemented in the region [12, 13]. While studies on the detection of asymptomatic, submicroscopic reservoirs and gametocyte carriers are becoming more common worldwide, none have been published from Mesoamerica. This study aimed to assess whether asymptomatic, submicroscopic reservoirs with gametocyte carriers exist in this region, to help guide novel control and elimination strategies in Mesoamerica.

## Methods

**Fig. 2:**
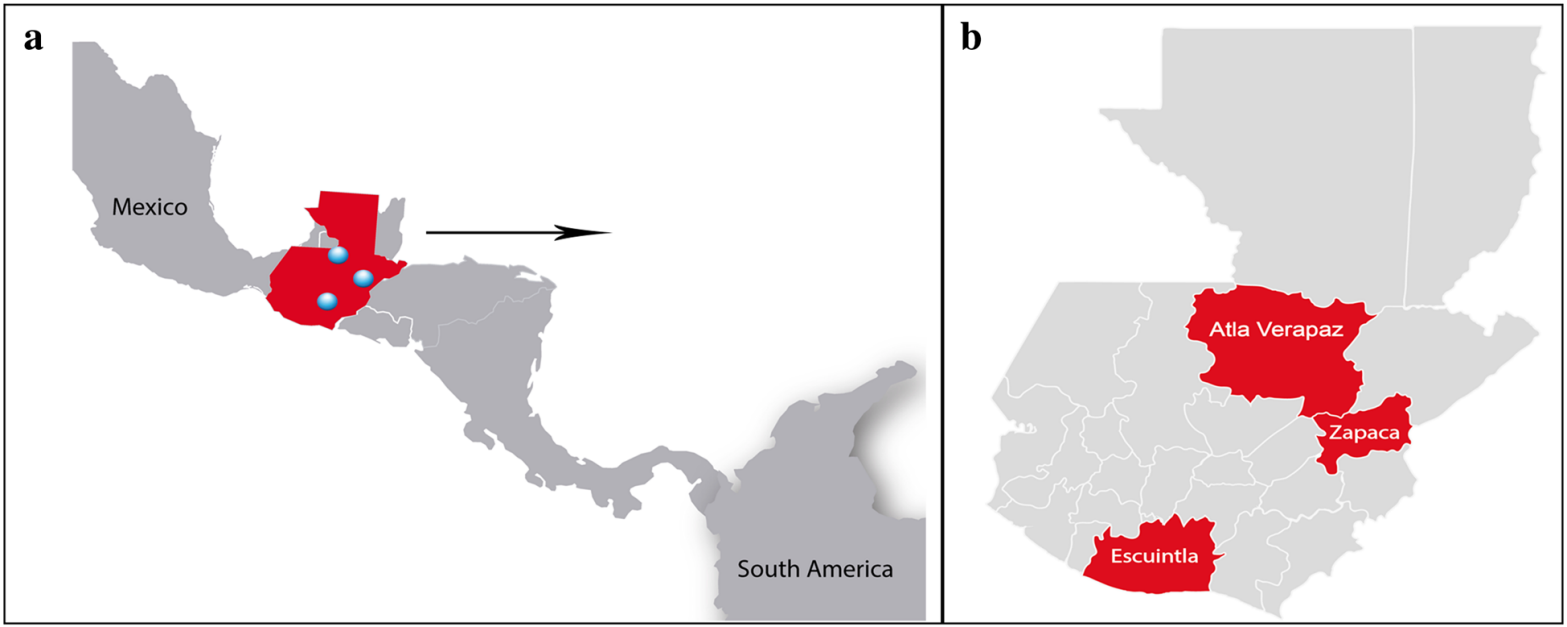
Study sites. The *three blue circles* in **a** show the location of the study sites in Guatemala and the departments (study sites) are enlarged in **b**

In May 2015, a cross-sectional study was performed across six sentinel sites (SSs) in three municipalities in three departments: Escuintla, Alta Verapaz and Zacapa (Fig. 2). Investigators went door to door in each location and recruited volunteers of all ages who were residing in the houses and agreed to sign informed consent forms. Blood samples were taken to determine malaria prevalence in each site and the proportion of gametocyte carriers.

### Study sites

**Fig. 3:**
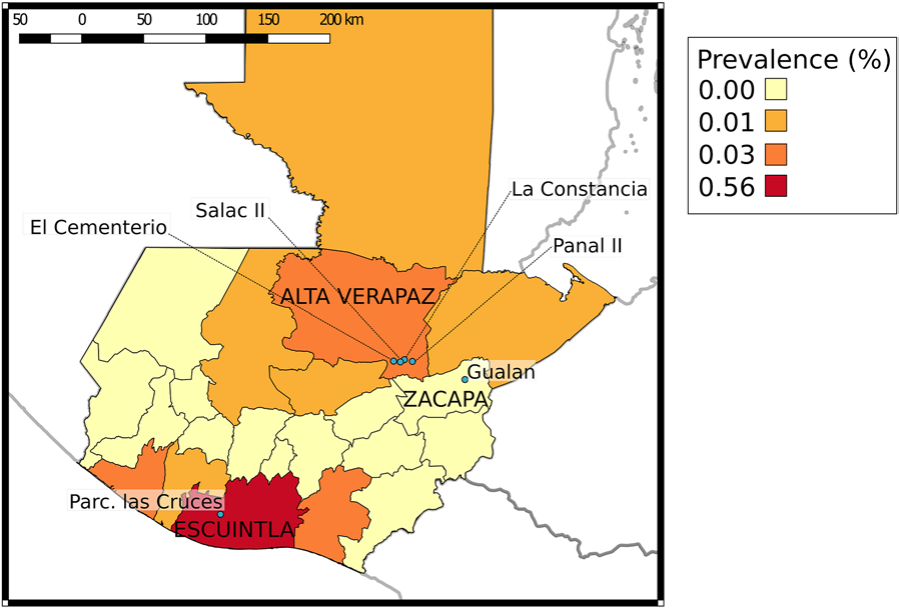
Map of study sentinel sites and malaria prevalences in 2015 per department. Guatemalan MOH surveillance data used

Three endemic study sites were chosen to include areas with differing transmission intensities. Parcelación de Las Cruces was the SS surveyed in the municipality of La Gomera in Escuintla, located in the south central region of Guatemala near the Pacific coast (Fig. 3). La Gomera, population 60,299, has a latitude and longitude of 14°08′31″ and −91°05′54″, respectively. At 136 m above sea level (m.a.s.l), it has an average annual temperature of 27.5 °C and rainfall of 1902 mm. It is mainly populated by mestizo people, who have both Hispanic and indigenous heritages and there is also a small indigenous population. The proportion of inhabitants living in urban and rural areas is similar; 51.1 and 48.9 %, respectively. From 2001 to 2013 the annual number of malaria cases increased by 92.6 % in Escuintla, reaching 3660 cases in 2013. Since 2013, a slight reduction in cases was observed with 2155 cases reported in 2015 (Guatemalan MOH surveillance data 2015) (Fig. 1b).

The four SSs, Panla II, El Cementerio, La Constancia, and Salac II, were surveyed in the municipality of La Tinta in Alta Verapaz. La Tinta, population 38,602, has a latitude and longitude of 15°31′22″ and −89°88′44″, respectively. At 195 m.a.s.l it has an average temperature of 27.9 °C and rainfall of 2448 mm. La Tinta is populated predominantly by indigenous people from various ethnic groups and has a tropical climate, which is similar to all other SSs. The department Alta Verapaz is predominantly rural (76.9 %) with only 23.1 % living in urban areas. The number of malaria cases in Alta Verapaz increased from 1059 in 2001 to 14,501 in 2006 when there was a malaria outbreak associated with a shortage of medication and medical personnel in remote areas. Since then, the overall trend has been a decline, with a second peak in 2012 and 218 cases reported in 2015 (Guatemalan MOH surveillance data 2015) (Fig. 1c).

Los Limones was the SS selected in Gualán (Zacapa) and has a population of 40,467. It is located in the eastern region of Guatemala and has a latitude and longitude of 15°11′95″ and −89°35′67″, respectively. At 130 m.a.s.l it has an average temperature of 27.1 °C and an annual rainfall of 1315 mm. It is populated predominantly by mestizo people and in the department of Zacapa there are slightly more inhabitants living in rural areas (57.4 %) than in urban areas (42.6 %). Between 2001 and 2008, Zacapa reported between zero and 29 cases of malaria annually. However, the prevalence started to increase thereafter and peaked at 1869 cases in 2010, when farm owners in Gualán did not give permission for vector program activities to be carried out. The number of cases subsequently decreased and zero cases were reported in 2012 and three in 2015 (Guatemalan MOH surveillance data 2015) (Fig. 1d).

The economies of all three departments rely heavily on farming and to a varying degree on livestock. In 2014, Escuintla had high malaria transmission, with greater than one case per 1000 population and Alta Verapaz and Zacapa had low malaria transmission with less than one case per 1000 population [1].

### Sample size

The sample size was calculated taking into account the population of each department studied and used a 95 % confidence interval with a margin of error of 3.6 % for Escuintla and 0.9 % for Alta Verapaz and Zacapa (due to lower reported malaria prevalence).

### Symptom surveys

Surveys were conducted to record each volunteer’s age, gender and malaria symptoms at the time of the survey. Symptom options included fever, chills, headache, profuse sweating, myalgia, and general malaise among others. Volunteers were asked if they had taken medication for their current symptoms, which medication they had taken and when their last malaria episode took place. An asymptomatic case of malaria was defined as an individual with a positive TBS and/or qPCR who did not have any symptoms.

### Malaria diagnostic tests

#### Thick blood smears (TBSs)

Approximately 100 µL of blood was collected by finger-prick and TBSs were prepared and stained by the Giemsa staining method for malaria diagnosis [14]. In positive films, parasite species were identified and the density was recorded as the number of parasites per 200 white blood cells. Two hundred high power fields of the thick blood films examined at 100× magnification were read before recording a negative result. Two microscopists read each slide separately and in cases of disagreement a third microscopist read the disputed slides and a sample of all the slides.

#### Venipuncture

Whole blood was drawn by venipuncture at the time of enrollment and collected in EDTA tubes (5 mL) for malaria parasite DNA isolation and qPCR and Tempus RNA tubes (3 mL, Thermofisher) for gametocyte determination. Venous blood samples collected were stored in polystyrene boxes containing frozen gel packs. They were then transported to the nearest health center in under 9 h at a temperature of 5 °C or less. Samples were centrifuged for sera and buffy coat separation. Fractions were frozen at temperatures between −15 and 25 °C and then shipped to Colombia in dry ice. qPCR was performed 1 week after arrival. Samples were handled as potential biohazards and all laboratory staff strictly followed standardized bio-safety procedures.

#### qPCR

Assays were performed as described previously [15, 16] with minor modifications. DNA was extracted from whole blood using the PureLink Genomic DNA kit. The amount of DNA in each well was adjusted to be equivalent to 1 μL of whole blood. Standard *P. falciparum* and *P. vivax* DNA positive and negative controls were used in each batch of tests including the extraction of both negative and inhibition controls. A sample was considered negative if there was no increase in the fluorescent signal after a minimum of 40 cycles. Parasitaemia quantification was performed using a parasite specific standard curve made with serial blood dilutions of a reference field isolate. Each reaction plate included a standard curve for parasite quantification. Positive samples were confirmed by an independent DNA extraction and a new round of qPCR by triplicates. Samples with at least two out of three positive wells in the confirmation reaction were considered positive. The Guatemalan health authorities were responsible for malaria treatment administration and received TBS and qPCR results within 3–4 weeks after the samples had been collected [21].

#### Gametocyte determination

RNA was extracted from the 200 µL aliquots conserved in Tempus RNA stabilization Buffer (Applied Biosystems, UK) using the PureLink RNA Mini Kit (Ambion, USA) following the manufacturer’s instructions. Purified RNA was added to 2 μL of DNase I and 2 μL of 10× Dnase I buffer which was then inactivated with 4 μL of Dnase inactivation reagent. A second round of Dnase digestion was performed to ensure genomic DNA removal. cDNA was obtained using a Super Script III Kit (Invitrogen, USA), according to the manufacturer’s instructions. Gene expression was performed in a 3-well by Real Time qPCR (7500 Real-Time PCR Systems; Applied Biosystems, USA) using SYBR Green and oligonucleotide primers as described previously [8]. Each reaction was performed in a total volume of 10 µL. To ensure DNA elimination, each sample was tested by qPCR targeting the housekeeping 18S rRNA gene of *P. vivax* in an independent reaction without reverse transcriptase. Sensitivity of this assay was determined using tenfold serial dilutions of cDNA as described elsewhere [8].

### Data entry, statistical analysis and quality assurance

Study data collected in the field were recorded on paper-based case report forms and digitalized using REDCap Version 6.9.4 (Research Electronic Data Capture). REDCap is a secure, web-based application designed to support data capture for research studies, providing: (1) an intuitive interface for validated data entry; (2) audit trails for tracking data manipulation and export procedures; (3) automated export procedures for seamless data downloads to common statistical packages and (4) procedures for importing data from external sources [17]. Once collected, data files were converted to MATLAB version 2014b (matrix laboratory) multi-paradigm numerical computing environment, using data export algorithm. All detected inconsistencies were resolved by correction against the original case report form or lab books [18].

Using R (a free software environment for statistical computing and graphics) [19] and MATLAB scripts and algorithms [20], a quality assurance process was performed to check for inconsistencies in the data, typing errors, missing data and outliers. Missing data was not included in the analyses. Statistical analyses were performed using Chi squared and Kruskal–Wallis tests (Additional file 1).

## Results

### Demographic features of study volunteers

The mean age of volunteers was 28, 24 and 25 years, in Escuintla, Alta Verapaz and Zacapa, respectively, and they were not significantly different (*W* = 2.4822, *p* = 0.29) (Table 1). As the study was carried out during the daytime (Monday to Saturday) it enrolled a significantly higher number of women in all three sites (66 %, *χ*^2^ = 72.346, *p* < 0.001). The number of volunteers in the 0–4 age group was significantly lower than in other age groups for all three sites (*p* < 0.001 controlling for the false discovery rate). However, there was no significant difference between the other age groups in each site.

**Table 1:** Demographic characteristics of study participants and qPCR results

Feature	Escuintla	Alta Verapaz	Zacapa	Overall
Sex	89/52	288/141	89/4	466/240
Number of females/males	*χ* ^2^ = 9.709 *p* = 0.0018	*χ* ^2^ = 50.371 *p* = 1.273 × 10^−12^	*χ* ^2^ = 12.971 *p* = 0.0003	*χ* ^2^ = 72.346 *p* < 2.2 × 10^−16^
Age	28 (20)	24 (17)	25 (18)	25 (18)
Mean (SD) Median (Range)	22 (2–86)	22** (0–99)	21 (3–99)	21 (0–99)
Number of volunteers per age group				
0–4	6	17	7	30
5–14	34	136	41	211
15–30	47	145	48	240
>30	54	130	40	224
Overall	141	428**	136	705**
P value (difference in age groups)	*χ* ^2^ = 38.206 *p* = 2.557 × 10^−08^	*χ* ^2^ = 102.000 *p* < 2.2 × 10^−16^	*χ* ^2^ = 29.706 *p* = 1.591 × 10^−06^	*χ* ^2^ = 378.970 *p* ≤ 2.2 × 10^−16^
Average parasitaemia (parasites/μl)	136.8 (351.6)	20.1 (25.2)	9.8 (8.2)	40.2 (153.6)
Mean (SD) Median (Range)	11 (1–1133)	10 (1–106)	7 (3–25)	9.5 (1–1133)
Number of asymptomatic cases in qPCR positive volunteers (%)	9/10 (90 %)	17/36 (47.2 %)	5/8 (72.5 %)	31/54 (57.4 %) *χ* ^2^ = 1.1852 *p* = 0.2763

*SD* standard deviation

** One age datum missing; *** W = 2.4822, *p* = 0.2891; difference in medians between sites

### Prevalence of *Plasmodium vivax* infections

**Fig. 4:**
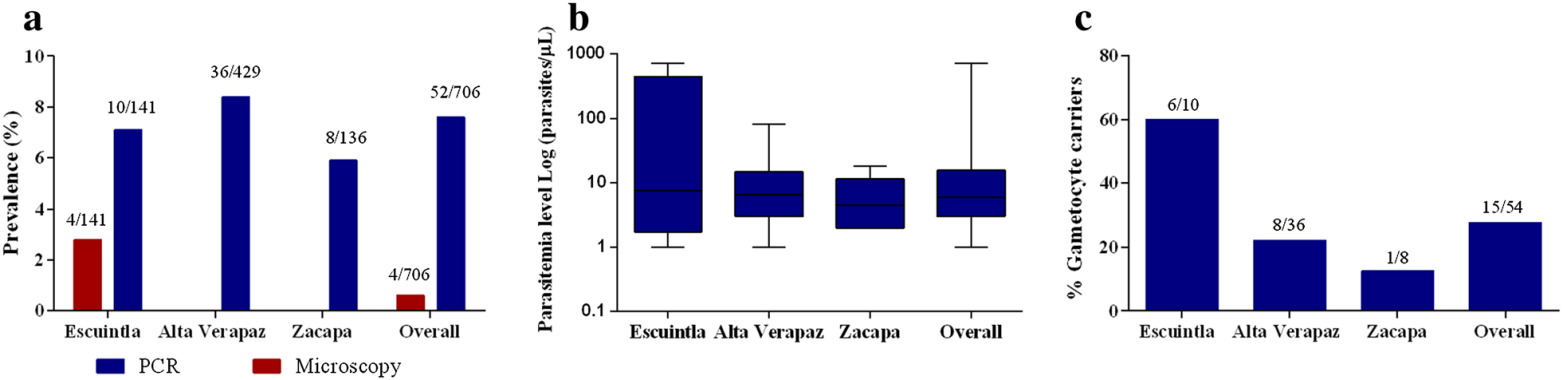
Results per study site and overall. **a** Prevalence of malaria by qPCR and microscopy, **b** parasitaemia levels, **c** percentage of mature gametocyte carriers in *P. vivax* infected individuals

All detected malaria infections were caused by *P. vivax.* The overall prevalence of malaria diagnosed by qPCR was 7.6 % (54/706, 95 % CI: 5.8–9.9), of which 7.4 % (4/54) were positive by TBS. qPCR positive individuals were distributed as follows: 7.1 % (10/141, CI: 3.6–13.0) in Escuintla, 8.4 % (36/429, CI: 2.8–11.6) in Alta Verapaz and 5.9 % (8/136, CI: 6.0–11.5) in Zacapa (Fig. 4a). The prevalence of malaria between the three sites was not significantly different (*χ*^2^ = 0.9977, *p* = 0.61). Microscopy detected malaria in Escuintla only in 2.8 % (4/141, CI: 0.9–7.6) of individuals tested, who were also positive by qPCR. This gave a false positive rate of zero (0/652) and a false negative rate of 92.6 % (50/54). The first microscopist reading was used in this analysis. The second microscopist detected two positive slides and a third microscopist detected three positive slides, all of which were detected by the first microscopist. An additional file shows the spread of malaria positive households in each study site (see Additional file 2). In Escuintla and Zacapa, cases were spread out, which contrasted to Alta Verapaz, where two clusters of three and six houses can be observed. Due to logistical problems, the planned number of volunteers was not recruited in Zacapa (136/434) and Alta Verapaz (429/430). In Zacapa, local leaders were unable to accompany the field workers in the field sites and they were therefore met with some suspicion by the target study population. This suspicion was heightened due to local elections which were taking place simultaneously. Potential volunteers, therefore, believed that field workers were affiliated with political parties. The impact of political events on conducting fieldwork, cultural beliefs and norms should not be overlooked when planning fieldwork.

Out of the 54 volunteers that tested positive for malaria with qPCR, 57.4 % (31/54) were asymptomatic (9/10 in Escuintla, 17/36 in Alta Verapaz and 5/8 in Zacapa). The remaining 42.6 % (23/54) were symptomatic, of which only one had a positive microscopy result. The other three individuals who had positive TBSs were asymptomatic.

In qPCR positive individuals, the prevalence of headache and fever was 52.2 % (12/23) and 39.1 % (9/23) respectively, whereas chills, profuse sweating, myalgia and general malaise were less prevalent (8.7 %, 2/23 for each symptom). Eleven of the 23 symptomatic individuals had malaria previously and their last episode was more than 1 month before the study (five more than 1 year and the other four reported episodes 1, 3, 4 and 7 months before). Two reported their last episode to have been 1 day ago, however, they had not taken any treatment. Eight of these volunteers had taken medication for their symptoms which included simple analgesia and three did not specify what they had taken. Thirteen of the 31 asymptomatic patients had malaria previously and their last episode was more than 1 month before the study (two had malaria 2 months previously, four had malaria 3 months previously, two had malaria 6 months previously and five did not answer/did not know). In qPCR negative volunteers, 28.9 % (188/651) were symptomatic, of which: 65.4 % (123/188) reported headache, 31.9 % (60/188) fever, 20.7 % (39/188) myalgia, 8.0 % (15/188) profuse sweating, 9.0 % (17/188) general malaise and 7.4 % (14/188) chills. No symptom data was available for one individual who had a negative qPCR result.

The average qPCR parasitaemia levels in Escuintla, Alta Verapaz and Zacapa were 136.8, 20.1 and 9.8 parasites/μL, respectively (Table 1; Fig. 4b). There were no significant differences in parasitaemia levels between the sites (*p* = 0.69, difference in medians calculated for non-normal data), nor between qPCR positive asymptomatic and symptomatic volunteers (*p* = 0.43). Additionally, no significant differences were found in parasitaemia levels overall when volunteers were grouped according to ages zero to four, five to 14, 15–30 and >30 years old (*χ*^2^ = 2.1248, *p* = 0.55).

### Mature gametocyte prevalence

Half (2/4) of the samples that were positive for malaria by microscopy were also positive for mature gametocytes and corresponded to asymptomatic volunteers. By contrast, 27.8 % (15/54) of the submicroscopic cases carried mature gametocytes. Of these, 86.7 % (13/15) were asymptomatic. The proportion of *P. vivax* gametocyte carriers was highest in Escuintla (60.0 %; 6/10), followed by Alta Verapaz (22.2 %; 8/36) and Zacapa (12.5 %; 1/8) (Fig. 4c).

## Discussion

This study was based on malaria ACD in three endemic regions with different transmission intensities in Guatemala. Escuintla had the highest number of malaria cases reported in the past decade, followed by Alta Verapaz and Zacapa. An overall malaria prevalence of 7.6 % was detected and similar prevalences were found in Alta Verapaz (8.4 %), Escuintla (7.1 %) and Zacapa (5.9 %). This was a striking observation as Zacapa is currently considered by the NMCP to be an area with an extremely low number of cases, whereas historically, Escuintla has had the highest transmission in the country (Guatemalan MOH surveillance data 2015) [21]. This study also found that 27.8 % of volunteers with malaria carried mature gametocytes and 86.7 % of those were asymptomatic, submicroscopic infections.

The potential for asymptomatic carriers to maintain transmission needs to be regarded as a critical public health problem as a proportion of these will harbour mature infected gametocytes that are likely capable of maintaining malaria transmission [5, 8, 22]. A Thai study found that 10 % (7/70) of *P. falciparum* infected volunteers and 13 % (7/52) of *P. vivax* volunteers (diagnosed by microscopy) infected *Anopheles dirus* mosquitoes using an artificial membrane feeding assay (MFA) [22]. Only 20 % of the volunteers with *P. falciparum* infections and 10 % with *P. vivax* infections were symptomatic. A Colombian study tested the infectivity of asymptomatic, submicroscopic *P. vivax* volunteers using MFAs and found that 57 % (8/14) of them were infective to *Anopheles albimanus* mosquitos, which was similar to the infection rate of symptomatic, submicroscopic, *P. vivax* volunteers [8]. In contrast, a Brazilian study found that 1.2 % of *Anopheles darlingi* mosquitoes became infected when they fed on volunteers with asymptomatic, submicroscopic infections (*P. vivax*, *P. falciparum* and mixed) [5]. However, the study authors argued that even though this was a low rate, asymptomatic infections remain undetected for longer than symptomatic infections. In this study one-quarter of volunteers with malaria carried mature gametocytes and the majority were asymptomatic and submicroscopic. Such infections have been considered of minor importance for control programmes, however they could be contributing significantly to the maintenance of malaria transmission.

*Plasmodium vivax* malaria is more challenging to eliminate than other species as it has a dormant stage, where hypnozoites hide in the liver and produce periodic relapses [10]. In this study at least 22.2 % of the volunteers reported having a previous malaria case between 1 and 7 months before the study (12/54) and we cannot rule out that these cases might have been relapses. Studies on hypnozoite diagnostic tests are imperative to diagnose these dormant phases [10]. In Guatemala the health authorities prescribe chloroquine and primaquine for *P. vivax* malaria and chloroquine for *P. falciparum* malaria. Once daily doses of 5 and 15 mg tablets of primaquine are given to children and adults, respectively for 14 days. Chloroquine is given over a course of 3 days to deliver 25 mg/kg in adults and in children, doses are prescribed according to their ages [23].

Only one of the 23 symptomatic qPCR positive volunteers had a positive microscopy result, which, as found previously, shows the potential for low parasitaemia levels to cause symptoms in *P. vivax* infections [24]. The number of symptomatic individuals (28.9 %) that were negative by both microscopy and qPCR was remarkable and suggests the presence of other fever syndromes in the region. Diseases such as dengue, leptospirosis, histoplasmosis and coccidioidomycosis have similar clinical presentations to malaria and may be under-diagnosed. Although this finding may also indicate a limited capacity of qPCR to detect malaria parasites in the blood volumes used here [25, 26], there is still a probable unmet need for more comprehensive studies to diagnose and manage other infectious febrile diseases in local health care services.

The majority of the infections found in this study would not have been detected by the current NMCP due to the low sensitivity of TBSs and RDTs that they use. This could lead them to conclude that malaria has been eliminated from regions such as Zacapa. The results show that hot spots existed in Alta Verapaz but not in Escuintla and Zacapa. The spread of malaria cases should be taken into account when deciding on the most suitable surveillance method.

Despite the low malaria transmission in Mesoamerica, Guatemala is considered one of the most endemic countries with 4931 cases in 2014, which contrasts with neighboring countries, such as El Salvador (eight cases) and Costa Rica (zero cases) [1]. However, the results reported here suggest that these countries, with only a few or zero annual cases reported, may be missing a great number of cases in the form of asymptomatic, submicroscopic infections due to diagnostic challenges. Furthermore, in the presence of efficient malaria vectors such as *Anopheles albimanus*, and *Anopheles vestitipennis* in the Mesoamerican region [11], the risk of malaria transmission resurgence is high, as it has been witnessed in other countries during elimination efforts [27]. Although periodic microscopist re-training would improve their skills to detect low parasitaemia levels, submicroscopic cases would remain undetected. Furthermore, the course and duration of asymptomatic infections is currently unknown [7], but may last for months or even years [9].

These study results are of utmost importance in view of the EMMIE initiative to bring malaria transmission to zero by 2020 [11]. Despite the additional costs that would be involved with the introduction of more sensitive molecular tests, their role in malaria elimination may prove cost-effective not only for Guatemala but also for other countries in the EMMIE initiative [28]. Furthermore, as shown in this study, the pattern of malaria cases varies in different communities. Therefore, preliminary cross-sectional surveys could be conducted before commencing control and elimination activities, to tailor them to each endemic setting.

## Conclusions

This study found a submicroscopic reservoir of *P. vivax* malaria in Guatemala with a high number of asymptomatic malaria cases and mature gametocytes. Malaria control and elimination programmes must be adapted to address changes in the presentation of malaria infections, with particular attention paid to parasite density and the appropriate diagnostic tests. Failure to do so may permit the perpetuation of malaria transmission through asymptomatic submicroscopic reservoirs [5, 7] or malaria outbreaks. Robust surveillance systems with molecular diagnostic tests are imperative to determine the true scale of this problem and to potentially prevent malaria resurgences. It is likely that there is no one-size-fits-all approach and case detection methods tailored to each endemic setting could prove effective to reduce the malaria burden. More research will provide evidence on the best methods and strategies for malaria elimination [6, 12].

## Additional files

10.1186/s12936-016-1500-6 Calculations booklet. This contains raw study data collected and the calculations performed for this manuscript.
10.1186/s12936-016-1500-6 Spread of malaria positive households per study site. This shows the shows the spread of malaria positive households in each study site.
