# Supplementary material for: Malaria elimination challenges in Mesoamerica: evidence of submicroscopic malaria reservoirs in Guatemala
Source: Malar J. 2016 Aug 30;15(1):441. doi: 10.1186/s12936-016-1500-6 (PMC5006524; doi:10.1186/s12936-016-1500-6)
Supplement: Supplementary file 1 — 10.1186/s12936-016-1500-6 Calculations booklet. This contains raw study data collected and the calculations performed for this manuscript. [file 12936_2016_1500_MOESM1_ESM.html]

Guatemala Paper 2015


# Guatemala Paper 2015

#### *Maria Isabel Arce*

#### *December 23- 2015*

Wtih a database named “mSympGuat” with the structure that gives the REDCap database design for Symptoms survey for the CLAIM P1.1 project, next is the procedure to obtain all the information from this data.

### Demographic information

```
#Total of samples
nrow(mSympGuat)
```

```
## [1] 706
```

```
#Samples by area
mSympGuat%>%group_by(sArea,sSite)%>%summarise(Total=n())
```

```
## Source: local data frame [3 x 3]
## Groups: sArea
## 
##          sArea                     sSite Total
## 1    Escuintla La_Gomera-Parc_Las_Cruces   141
## 2       Zacapa                    Gualan   136
## 3 Alta_Verapaz                  La_tinta   429
```

```
#Ages by area
mStatsAgeByArea<-mSympGuat %>% filter(!is.na(no_age)) %>%group_by(sArea)%>% 
  summarise(mean=round(mean(no_age)),sd=round(sd(no_age)),median=median(no_age)*1.0,min=min(no_age),max=max(no_age)) ; mStatsAgeByArea
```

```
## Source: local data frame [3 x 6]
## 
##          sArea mean sd median min max
## 1    Escuintla   28 20     22   2  86
## 2       Zacapa   25 18     21   3  99
## 3 Alta_Verapaz   24 17     22   0  99
```

```
#Differences between ages by site
shapiro.test(mSympGuat$no_age)
```

```
## 
##  Shapiro-Wilk normality test
## 
## data:  mSympGuat$no_age
## W = 0.90415, p-value < 2.2e-16
```

```
kruskal.test(mSympGuat$no_age~mSympGuat$sArea)
```

```
## 
##  Kruskal-Wallis rank sum test
## 
## data:  mSympGuat$no_age by mSympGuat$sArea
## Kruskal-Wallis chi-squared = 2.4822, df = 2, p-value = 0.2891
```

There’s no differences between median ages between areas.

The proportion of males and females are statistically different. With proportion of females larger than males (The same for each area).

```
#Gender clasification
mSexGuat<-mSympGuat  %>%group_by(Sex=sSex)  %>%summarise(Cant=n(), Prop=round(Cant*100/nrow(mSympGuat)));mSexGuat
```

```
## Source: local data frame [2 x 3]
## 
##      Sex Cant Prop
## 1   Male  240   34
## 2 Female  466   66
```

```
chisq.test(mSexGuat[,2])
```

```
## 
##  Chi-squared test for given probabilities
## 
## data:  mSexGuat[, 2]
## X-squared = 72.346, df = 1, p-value < 2.2e-16
```

```
mSexGuatArea<-mSympGuat  %>%group_by(Sex=sSex)  %>%summarise(Escuintla=sum(sArea=="Escuintla"),Alta_Verapaz=sum(sArea=="Alta_Verapaz"),Zacapa=sum(sArea=="Zacapa"));mSexGuatArea
```

```
## Source: local data frame [2 x 4]
## 
##      Sex Escuintla Alta_Verapaz Zacapa
## 1   Male        52          141     47
## 2 Female        89          288     89
```

```
chisq.test(mSexGuatArea[,2]) #Escuintla
```

```
## 
##  Chi-squared test for given probabilities
## 
## data:  mSexGuatArea[, 2]
## X-squared = 9.7092, df = 1, p-value = 0.001833
```

```
chisq.test(mSexGuatArea[,3]) #Alta Verapaz
```

```
## 
##  Chi-squared test for given probabilities
## 
## data:  mSexGuatArea[, 3]
## X-squared = 50.371, df = 1, p-value = 1.273e-12
```

```
chisq.test(mSexGuatArea[,4]) #Zacapa
```

```
## 
##  Chi-squared test for given probabilities
## 
## data:  mSexGuatArea[, 4]
## X-squared = 12.971, df = 1, p-value = 0.0003164
```

### Age Groups

```
#For Guatemala
mGuatAgeG<-mSympGuat%>%group_by(nAge)%>%summarise(n=n());mGuatAgeG
```

```
## Source: local data frame [5 x 2]
## 
##    nAge   n
## 1   0-4  30
## 2  5-14 211
## 3 15-30 240
## 4   >30 224
## 5    NA   1
```

```
chisq.test(mGuatAgeG[,2])
```

```
## 
##  Chi-squared test for given probabilities
## 
## data:  mGuatAgeG[, 2]
## X-squared = 378.97, df = 4, p-value < 2.2e-16
```

```
#One case without age excluded of the rest of the analysis
p<-c(chisq.test(mGuatAgeG[1:2,2])[3],chisq.test(mGuatAgeG[c(1,3),2])[3],chisq.test(mGuatAgeG[c(1,4),2])[3],chisq.test(mGuatAgeG[c(2,3),2])[3],chisq.test(mGuatAgeG[c(2,4),2])[3],chisq.test(mGuatAgeG[c(3,4),2])[3])
pad<-p.adjust(p,method="fdr",n=length(p));names(pad)<-c("p_0-4Vs5-14","p_0-4Vs15-30","p_0-4Vs>30","p_5-14Vs15-30","p_5-14Vs>30","p_15-30Vs>30");pad
```

```
##   p_0-4Vs5-14  p_0-4Vs15-30    p_0-4Vs>30 p_5-14Vs15-30   p_5-14Vs>30 
##  4.117680e-31  1.269263e-36  1.304324e-33  2.581172e-01  5.330859e-01 
##  p_15-30Vs>30 
##  5.330859e-01
```

The proportion of age groups in Guatemala for each area:

```
#For each area
mGuatAreaAgeG<-mSympGuat%>%group_by(nAge)%>%summarise(Escuintla=sum(sArea=="Escuintla"),Alta_Verapaz=sum(sArea=="Alta_Verapaz"),Zacapa=sum(sArea=="Zacapa"));mGuatAreaAgeG
```

```
## Source: local data frame [5 x 4]
## 
##    nAge Escuintla Alta_Verapaz Zacapa
## 1   0-4         6           17      7
## 2  5-14        34          136     41
## 3 15-30        47          145     48
## 4   >30        54          130     40
## 5    NA         0            1      0
```

The age group between 0-4 was statistically different from the other age groups. The same for each area.

The proportion of age groups for each area. Escuintla:

```
chisq.test(mGuatAreaAgeG[1:4,2])#Escuintla
```

```
## 
##  Chi-squared test for given probabilities
## 
## data:  mGuatAreaAgeG[1:4, 2]
## X-squared = 38.206, df = 3, p-value = 2.557e-08
```

```
p<-c(chisq.test(mGuatAreaAgeG[1:2,2])[3],chisq.test(mGuatAreaAgeG[c(1,3),2])[3],chisq.test(mGuatAreaAgeG[c(1,4),2])[3],chisq.test(mGuatAreaAgeG[2:3,2])[3],chisq.test(mGuatAreaAgeG[c(2,4),2])[3],chisq.test(mGuatAreaAgeG[3:4,2])[3])
pad<-p.adjust(p,method="fdr",n=length(p));names(pad)<-c("p_0-4Vs5-14","p_0-4Vs15-30","p_0-4Vs>30","p_5-14Vs15-30","p_5-14Vs>30","p_15-30Vs>30");pad
```

```
##   p_0-4Vs5-14  p_0-4Vs15-30    p_0-4Vs>30 p_5-14Vs15-30   p_5-14Vs>30 
##  1.909384e-05  5.350677e-08  3.457944e-09  1.783368e-01  4.950939e-02 
##  p_15-30Vs>30 
##  4.860995e-01
```

Alta Verapaz:

```
chisq.test(mGuatAreaAgeG[1:4,3])#Alta Verapaz
```

```
## 
##  Chi-squared test for given probabilities
## 
## data:  mGuatAreaAgeG[1:4, 3]
## X-squared = 102, df = 3, p-value < 2.2e-16
```

```
p<-c(chisq.test(mGuatAreaAgeG[1:2,3])[3],chisq.test(mGuatAreaAgeG[c(1,3),3])[3],chisq.test(mGuatAreaAgeG[c(1,4),3])[3],chisq.test(mGuatAreaAgeG[2:3,3])[3],chisq.test(mGuatAreaAgeG[c(2,4),3])[3],chisq.test(mGuatAreaAgeG[3:4,3])[3])
pad<-p.adjust(p,method="fdr",n=length(p));names(pad)<-c("p_0-4Vs5-14","p_0-4Vs15-30","p_0-4Vs>30","p_5-14Vs15-30","p_5-14Vs>30","p_15-30Vs>30");pad
```

```
##   p_0-4Vs5-14  p_0-4Vs15-30    p_0-4Vs>30 p_5-14Vs15-30   p_5-14Vs>30 
##  1.963834e-21  5.153301e-23  2.324988e-20  7.096082e-01  7.129600e-01 
##  p_15-30Vs>30 
##  5.485684e-01
```

Zacapa:

```
chisq.test(mGuatAreaAgeG[1:4,4])#Zacapa
```

```
## 
##  Chi-squared test for given probabilities
## 
## data:  mGuatAreaAgeG[1:4, 4]
## X-squared = 29.706, df = 3, p-value = 1.591e-06
```

```
p<-c(chisq.test(mGuatAreaAgeG[1:2,4])[3],chisq.test(mGuatAreaAgeG[c(1,3),4])[3],chisq.test(mGuatAreaAgeG[c(1,4),4])[3],chisq.test(mGuatAreaAgeG[2:3,4])[3],chisq.test(mGuatAreaAgeG[c(2,4),4])[3],chisq.test(mGuatAreaAgeG[3:4,4])[3])
pad<-p.adjust(p,method="fdr",n=length(p));names(pad)<-c("p_0-4Vs5-14","p_0-4Vs15-30","p_0-4Vs>30","p_5-14Vs15-30","p_5-14Vs>30","p_15-30Vs>30");pad
```

```
##   p_0-4Vs5-14  p_0-4Vs15-30    p_0-4Vs>30 p_5-14Vs15-30   p_5-14Vs>30 
##  2.767660e-06  1.938558e-07  2.965528e-06  5.497059e-01  9.115282e-01 
##  p_15-30Vs>30 
##  5.497059e-01
```

### Prevalences

The total prevalence for guatemala was:

```
mPCRPosiYearGuat<-mSympGuat%>%filter(nm_result_pcr==1)%>%group_by(Year=nYear)%>%summarise(Posi=sum(nm_result_pcr),PosiMic=sum(nResultTbs))
mSampleYearGuat<-mSympGuat%>%group_by(Year=nYear)%>%summarise(Total=n())
mPCRPosiYearGuat$nTotal<-mSampleYearGuat$Total
mPCRPosiYearGuat$nPercent<-round(mPCRPosiYearGuat$Posi*100/mPCRPosiYearGuat$nTotal,1)
mPCRPosiYearGuat$nPercentMic<-round(mPCRPosiYearGuat$PosiMic*100/mPCRPosiYearGuat$nTotal,1);mPCRPosiYearGuat
```

```
## Source: local data frame [1 x 6]
## 
##   Year Posi PosiMic nTotal nPercent nPercentMic
## 1 2015   54       4    706      7.6         0.6
```

```
#Confidence intervals
round(prop.test(as.numeric(mPCRPosiYearGuat[2]),as.numeric(mPCRPosiYearGuat[4]))[6]$conf.int*100,1)
```

```
## [1] 5.8 9.9
## attr(,"conf.level")
## [1] 0.95
```

Prevalences by area as follow:

```
mPCRPosiYearAreaGuat<-mSympGuat%>%filter(nm_result_pcr==1)%>%group_by(Year=nYear,Area=sArea)%>%summarise(Posi=sum(nm_result_pcr),PosiMic=sum(nResultTbs))
mSampleYearAreaGuat<-mSympGuat%>%group_by(Year=nYear,Area=sArea)%>%summarise(Total=n())
mPCRPosiYearAreaGuat$nTotal<-mSampleYearAreaGuat$Total
mPCRPosiYearAreaGuat$nPercent<-round(mPCRPosiYearAreaGuat$Posi*100/mPCRPosiYearAreaGuat$nTotal,1)
mPCRPosiYearAreaGuat$nPercentMic<-round(mPCRPosiYearAreaGuat$PosiMic*100/mPCRPosiYearAreaGuat$nTotal,1)

mPCRPosiYearAreaGuat$CI_Inf<-round(c(prop.test(10,141)[6]$conf.int[1]*100,prop.test(8,136)[6]$conf.int[1]*100 ,prop.test(36,429)[6]$conf.int[1]*100),1)
mPCRPosiYearAreaGuat$CI_Sup<-round(c(prop.test(10,141)[6]$conf.int[2]*100,prop.test(8,136)[6]$conf.int[2]*100 ,prop.test(36,429)[6]$conf.int[2]*100),1);mPCRPosiYearAreaGuat
```

```
## Source: local data frame [3 x 9]
## Groups: Year
## 
##   Year         Area Posi PosiMic nTotal nPercent nPercentMic CI_Inf CI_Sup
## 1 2015    Escuintla   10       4    141      7.1         2.8    3.6   13.0
## 2 2015       Zacapa    8       0    136      5.9         0.0    2.8   11.6
## 3 2015 Alta_Verapaz   36       0    429      8.4         0.0    6.0   11.5
```

```
#Test for diferences
chisq.test(cbind(mPCRPosiYearAreaGuat[,c(3)],mPCRPosiYearAreaGuat[,5]-mPCRPosiYearAreaGuat[,3]))
```

```
## 
##  Pearson's Chi-squared test
## 
## data:  cbind(mPCRPosiYearAreaGuat[, c(3)], mPCRPosiYearAreaGuat[, 5] -     mPCRPosiYearAreaGuat[, 3])
## X-squared = 0.99771, df = 2, p-value = 0.6072
```

### Asymptomatic

```
mSymptoPCRSpP11Guat<-mSympGuat %>% group_by(Symptom=in_have_symptom,PCR_result=sPcrResult) %>% summarise(n=n())%>%mutate(per=round(n*100/706,1));mSymptoPCRSpP11Guat
```

```
## Source: local data frame [5 x 4]
## Groups: Symptom
## 
##   Symptom PCR_result   n  per
## 1       0       PCR+  31  4.4
## 2       0       PCR- 463 65.6
## 3       1       PCR+  23  3.3
## 4       1       PCR- 188 26.6
## 5      NA       PCR-   1  0.1
```

```
mSymptoSpP11Guat<-mSympGuat %>% filter(nm_result_pcr==1 ) %>% group_by(Symptom=in_have_symptom,Plasmodium=sParasiteSp) %>% summarise(n=n())%>%mutate(per=round(n*100/54,1));mSymptoSpP11Guat
```

```
## Source: local data frame [2 x 4]
## Groups: Symptom
## 
##   Symptom Plasmodium  n  per
## 1       0   P. vivax 31 57.4
## 2       1   P. vivax 23 42.6
```

```
chisq.test(mSymptoSpP11Guat[,3])
```

```
## 
##  Chi-squared test for given probabilities
## 
## data:  mSymptoSpP11Guat[, 3]
## X-squared = 1.1852, df = 1, p-value = 0.2763
```

```
#Asymptomtics by area
mAsymByArea<-mSympGuat %>%filter(in_have_symptom==0) %>% group_by(PCR_result=sPcrResult,Area=sArea) %>% summarise(n=n());mAsymByArea
```

```
## Source: local data frame [6 x 3]
## Groups: PCR_result
## 
##   PCR_result         Area   n
## 1       PCR+    Escuintla   9
## 2       PCR+       Zacapa   5
## 3       PCR+ Alta_Verapaz  17
## 4       PCR-    Escuintla 127
## 5       PCR-       Zacapa  98
## 6       PCR- Alta_Verapaz 238
```

No significant differences were found between PCR result and area.

### Parasitemias

Summary statistics for parasitemia data overall and by site:

```
mSympGuat %>%summarise(n=n(),median=median(no_parasitemia_vivax,na.rm=T),sd=sd(no_parasitemia_vivax,na.rm=T),mean=mean(no_parasitemia_vivax,na.rm=T),min=min(no_parasitemia_vivax,na.rm=T),max=max(no_parasitemia_vivax,na.rm=T))
```

```
##     n median       sd     mean min  max
## 1 706    9.5 153.6113 40.18519   1 1133
```

```
mSympGuat$nParasitemia<-mSympGuat$no_parasitemia_vivax;mSympGuat$nParasitemia[complete.cases(mSympGuat$no_parasitemia_falciparum)]<-mSympGuat$no_parasitemia_falciparum[complete.cases(mSympGuat$no_parasitemia_falciparum)]
mParasitemiaPvP11Guat<-mSympGuat %>%filter(!is.na(no_parasitemia_vivax)) %>% group_by(sArea) %>%summarise(mean=round(mean(no_parasitemia_vivax),3),sd=round(sd(no_parasitemia_vivax),3),median=round(median(no_parasitemia_vivax),3),min=min(no_parasitemia_vivax),max=max(no_parasitemia_vivax));mParasitemiaPvP11Guat
```

```
## Source: local data frame [3 x 6]
## 
##          sArea    mean      sd median min  max
## 1    Escuintla 136.800 351.605     11   1 1133
## 2       Zacapa   9.750   8.155      7   3   25
## 3 Alta_Verapaz  20.111  25.212     10   1  106
```

```
kruskal.test(mSympGuat$no_parasitemia_vivax,mSympGuat$sArea)
```

```
## 
##  Kruskal-Wallis rank sum test
## 
## data:  mSympGuat$no_parasitemia_vivax and mSympGuat$sArea
## Kruskal-Wallis chi-squared = 0.74964, df = 2, p-value = 0.6874
```

```
#By Age
mSympGuat %>%group_by(nAge)%>%summarise(n=n(),median=median(no_parasitemia_vivax,na.rm=T),min=min(no_parasitemia_vivax,na.rm=T),max=max(no_parasitemia_vivax,na.rm=T))
```

```
## Source: local data frame [5 x 5]
## 
##    nAge   n median min  max
## 1   0-4  30   20.0  14   26
## 2  5-14 211    9.5   1 1133
## 3 15-30 240   10.0   1  106
## 4   >30 224    6.0   2   40
## 5    NA   1     NA  NA   NA
```

```
kruskal.test(mSympGuat$no_parasitemia_vivax,mSympGuat$nAge)
```

```
## 
##  Kruskal-Wallis rank sum test
## 
## data:  mSympGuat$no_parasitemia_vivax and mSympGuat$nAge
## Kruskal-Wallis chi-squared = 2.1248, df = 3, p-value = 0.5469
```

### Symptoms

Symptoms reported were:

```
mSymptomsP11Guat<-funSumSymptoms(mSympGuat);mSymptomsP11Guat
```

```
## Source: local data frame [2 x 10]
## 
##   sPcrResult Fever Chills Headache Profuse_sweating Muscle_pain Malaise
## 1       PCR+     9      2       12                2           2       2
## 2       PCR-    60     14      123               15          39      17
## Variables not shown: All_previous (dbl), None (dbl), Other (int)
```

```
#percentage of symptoms for people with PCR+
mSympTableP11Guat<-round(mSymptomsP11Guat[1,-1]*100/sum(na.exclude(mSymptoPCRSpP11Guat$n[mSymptoPCRSpP11Guat$PCR_result=="PCR+"])));mSympTableP11Guat
```

```
##   Fever Chills Headache Profuse_sweating Muscle_pain Malaise All_previous
## 1    17      4       22                4           4       4            0
##   None Other
## 1    0     7
```

```
#percentage of symptoms for people with PCR-
round(mSymptomsP11Guat[2,-1]*100/sum(na.exclude(mSymptoPCRSpP11Guat$n[mSymptoPCRSpP11Guat$PCR_result=="PCR-"])))
```

```
##   Fever Chills Headache Profuse_sweating Muscle_pain Malaise All_previous
## 1     9      2       19                2           6       3            0
##   None Other
## 1    0     3
```
