# Supplementary figures and images for: Malaria elimination challenges in Mesoamerica: evidence of submicroscopic malaria reservoirs in Guatemala
Source: Malar J. 2016 Aug 30;15(1):441. doi: 10.1186/s12936-016-1500-6 (PMC5006524; doi:10.1186/s12936-016-1500-6)

## ALTA VERAPAZ

20 0 20 40 60 80 m

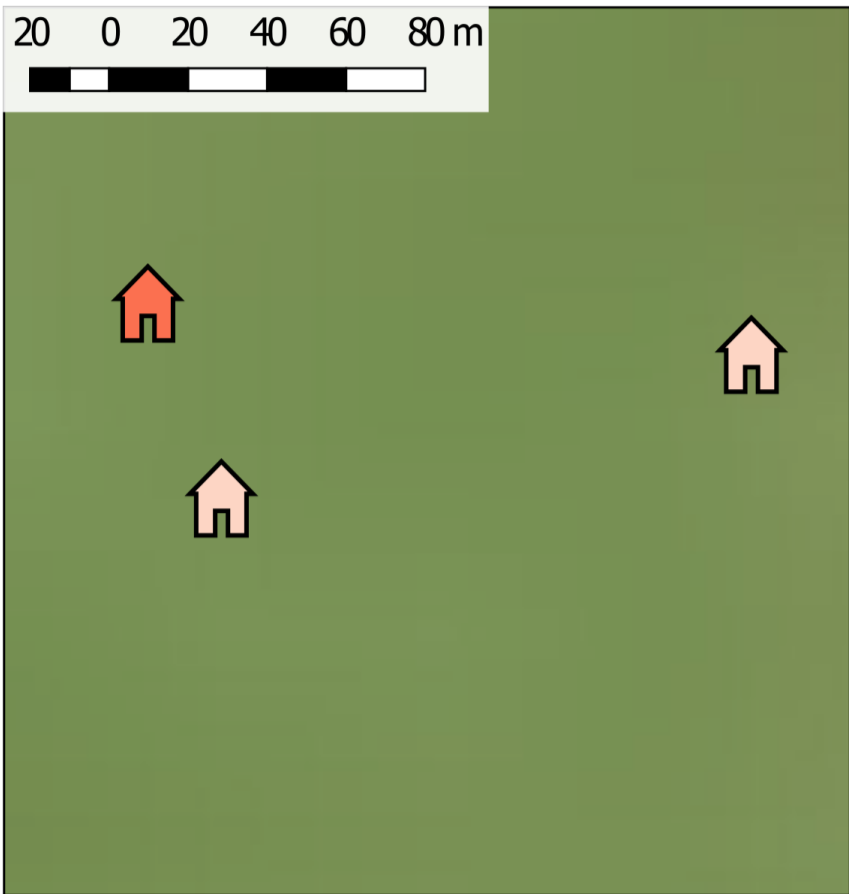

20 0 20 40 60 80 m

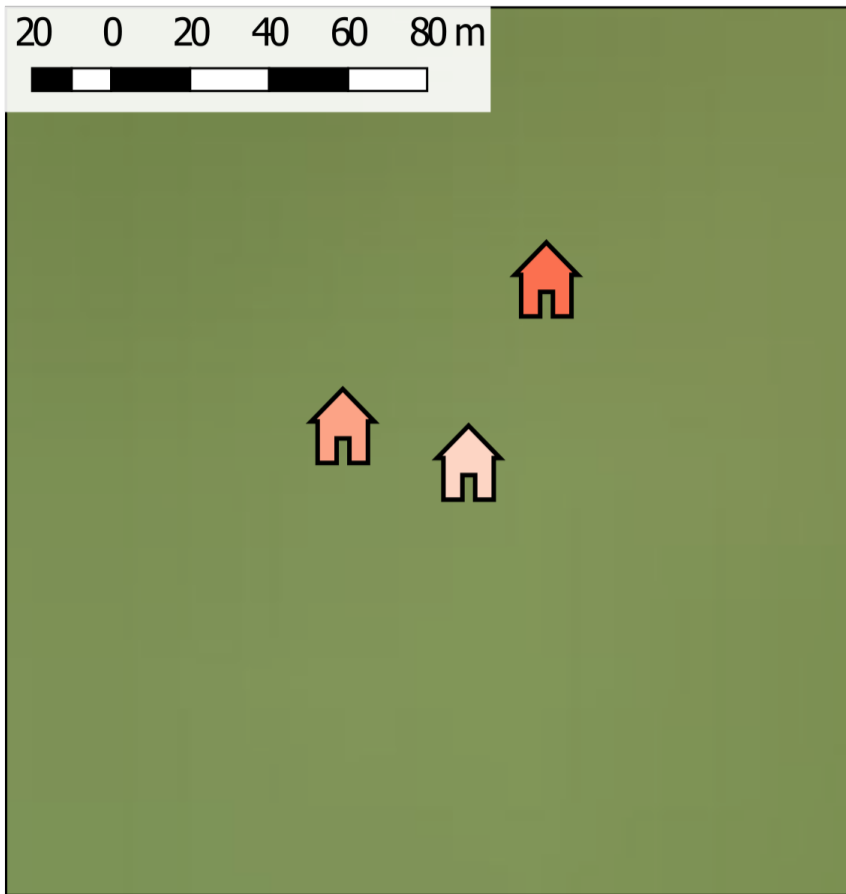

## GUATEMALA

25 0 25 50 75 100 km

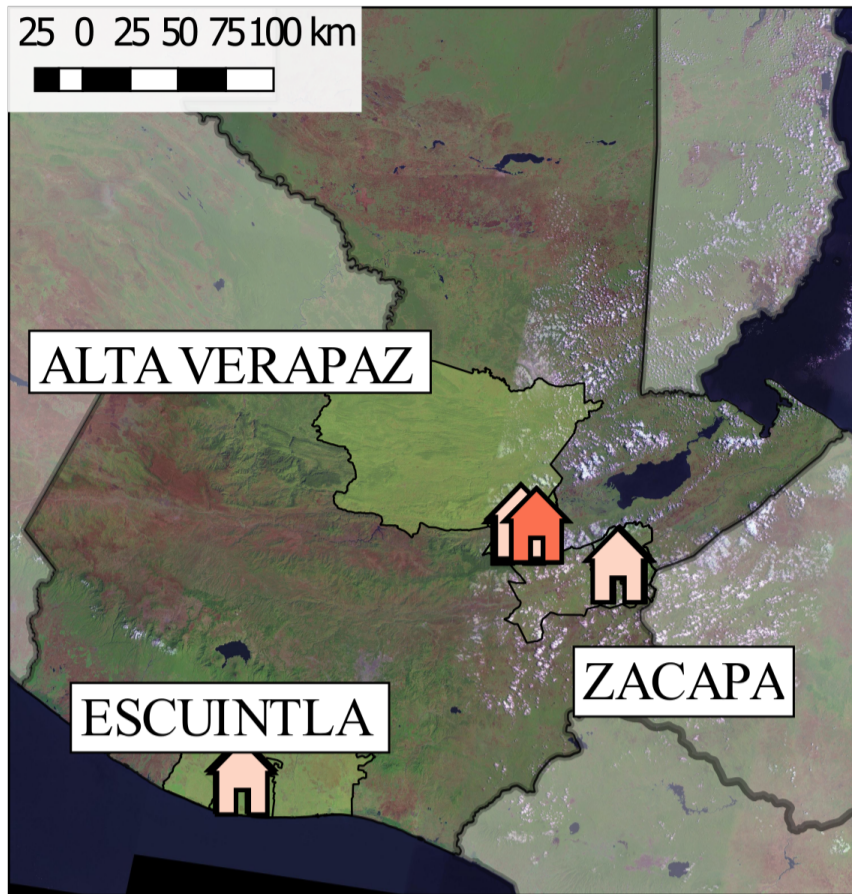

## ZACAPA

100 0 100 200 300 400 m

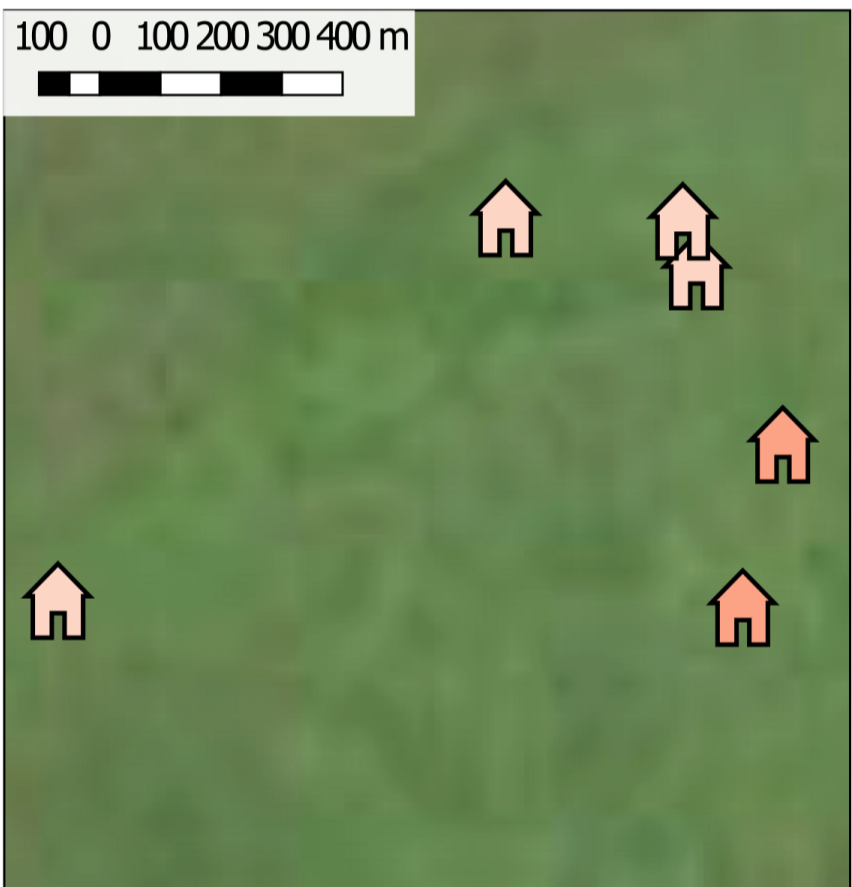

## ESCUINTLA

10 0 10 20 30 40 m

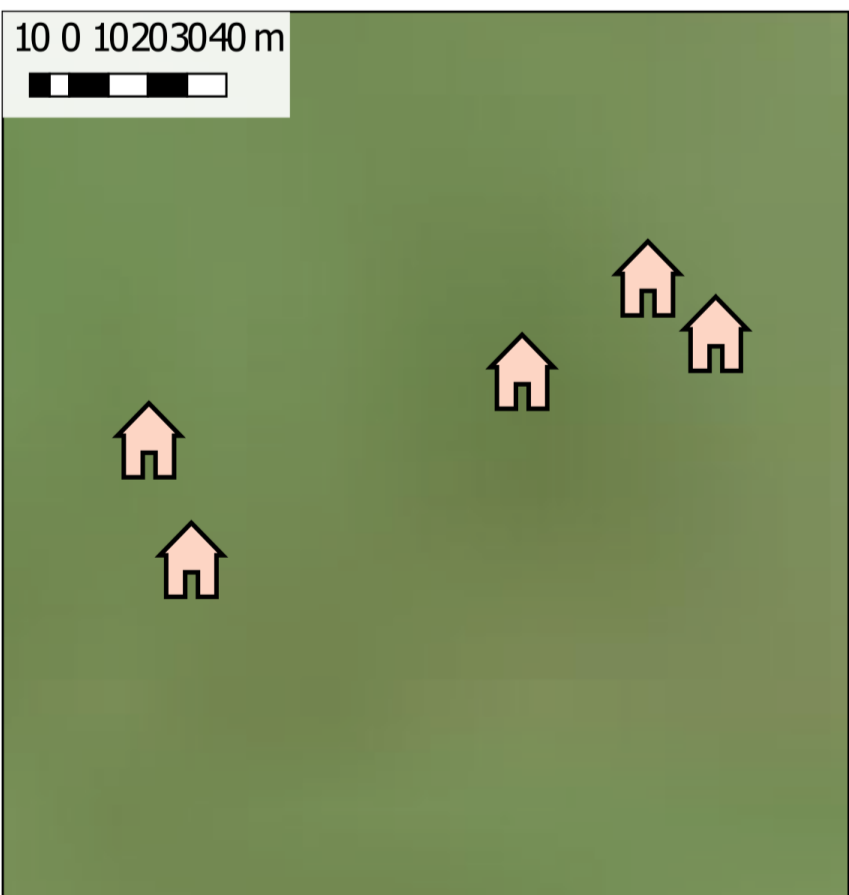

10 0 10 20 30 40 m

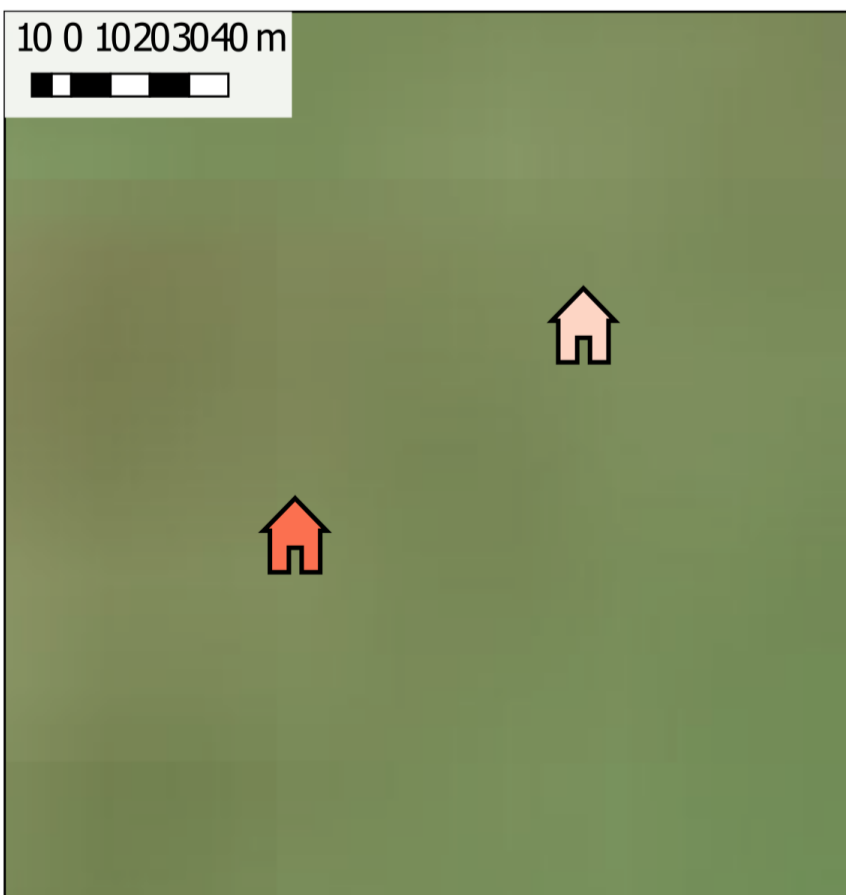

10 0 10 20 30 40 m

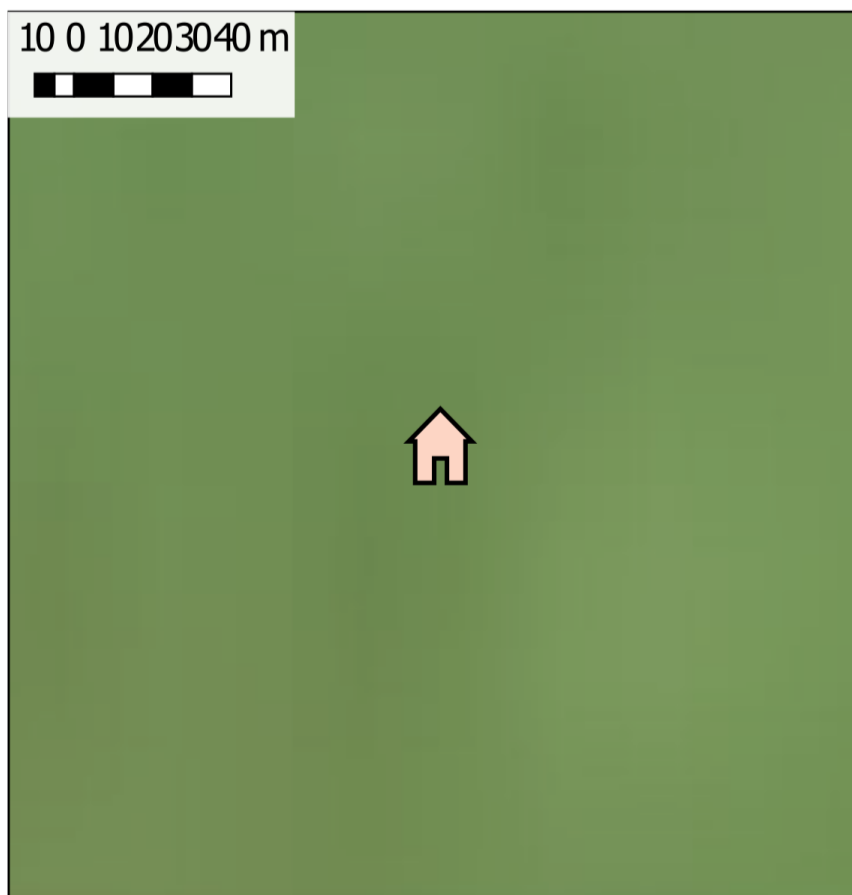

### Malaria Cases

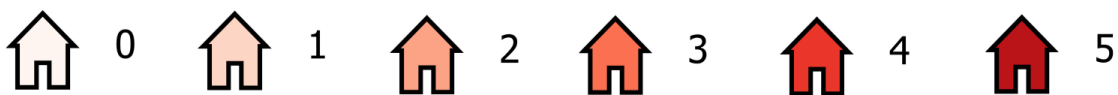

Supplement: Supplementary file 2 — 10.1186/s12936-016-1500-6 Spread of malaria positive households per study site. This shows the shows the spread of malaria positive households in each study site. [file 12936_2016_1500_MOESM2_ESM.pdf]
